# miR-3174 Is a New Tumor Suppressor MicroRNA That Inhibits Several Tumor-Promoting Genes in Glioblastoma

**DOI:** 10.3390/ijms24119326

**Published:** 2023-05-26

**Authors:** Farina Hanif, Ying Zhang, Collin Dube, Myron K. Gibert, Shekhar Saha, Kadie Hudson, Pawel Marcinkiewicz, Benjamin Kefas, Fadila Guessous, Roger Abounader

**Affiliations:** 1 Department of Microbiology, Immunology, and Cancer Biology, School of Medicine, University of Virginia, Charlottesville, VA 22908, USA; farina.hanif@duhs.edu.pk (F.H.); yz5h@virginia.edu (Y.Z.); cjd7ua@virginia.edu (C.D.); mkg7x@virginia.edu (M.K.G.J.); ss7st@virginia.edu (S.S.); rce3ka@virginia.edu (K.H.); pmarcinkiewicz98@outlook.com (P.M.); bak4x@hscmail.mcc.virginia.edu (B.K.); guessousf@hotmail.com (F.G.); 2 Department of Biochemistry, Dow International Medical College, Dow University of Health Sciences, OJHA Campus, SUPARCO Road, Karachi 74200, Pakistan; 3 University of Virginia Comprehensive Cancer Center, Charlottesville, VA 22908, USA; 4 Department of Neurology, School of Medicine, University of Virginia, Charlottesville, VA 22908, USA

**Keywords:** glioblastoma, microRNA, miR-3174, CDK6, Cd44, MDM2, PLAU, RHOA

## Abstract

microRNAs (miRNAs) play an important role in the pathology of glioblastoma (GBM), which is the most malignant and most common primary malignant brain tumor. miRNAs can target multiple genes simultaneously and are considered as potential therapeutic agents or targets. This study aimed to determine the role of miR-3174 in the pathobiology of GBM using both in vitro and in vivo approaches. This is the first study deciphering the role of miR-3174 in GBM. We studied the expression of miR-3174 and found it to be downregulated in a panel of GBM cell lines, GSCs and tissues relative to astrocytes and normal brain tissue. This finding led us to hypothesize that miR-3174 has a tumor-suppressive role in GBM. Exogenous expression of miR-3174 inhibited GBM cell growth and invasion, and hampered the neurosphere formation ability of GSCs. miR-3174 downregulated the expression of multiple tumor-promoting genes including CD44, MDM2, RHOA, PLAU and CDK6. Further, overexpression of miR-3174 reduced tumor volume in nude mice with intracranial xenografts. Immuno-histochemical study of brain sections with intracranial tumor xenografts revealed the pro-apoptotic and anti-proliferative activity of miR-3174. In conclusion, we demonstrated that miR-3174 has a tumor-suppressive role in GBM and could be exploited for therapeutic purposes.

## 1. Introduction

Non-coding RNAs (ncRNAs) are a class of molecules which have been under the limelight since their discovery because of their diverse and extensive role in maintaining and regulating cellular functions [1]. microRNAs play very important roles in various physiological and pathological processes, as they regulate the expression of many genes by binding to the 3′ untranslated region (3′ UTR) of the target mRNA, leading to either inhibition of mRNA translation or its degradation [2,3]. Many studies have reported the role of miRNAs in various malignancies including glioblastoma (GBM) [3,4,5,6]. GBM is the most malignant primary brain tumor, notorious for having a dismal prognosis [7]. Despite the advancement in chemo/radiation therapy and the availability of cutting-edge surgical modalities, the prognosis has not significantly improved [8]. Molecular heterogeneity, glioblastoma stem cells (GSCs), multiple deregulated cell signaling pathways and capacity of glioblastoma cells to infiltrate the surrounding tissue are considered the main reasons for the therapeutic resistance/treatment failure [9,10]. As single miRNAs are known to target multiple genes at the same time and hence target multiple pathways they are considered as potential therapeutic agents or targets in GBM [11]. 

Various studies published in the past have reported the roles of different miRNAs in regulating all major hallmarks of cancer including angiogenesis [12,13], uncontrolled proliferation [3,14,15], resistance to apoptosis and escape from the immune system [16]. Many miRNAs are deregulated in GBM [14,15,17]. They can serve as either tumor suppressors or oncogenes (onco-miRs). Many tumor-suppressive miRNAs are downregulated in GBM while the opposite is true for the onco-miRs, where their upregulation downregulates the expression of many tumor-suppressor genes and hence enhances tumor malignancy. Therefore, overexpression of tumor-suppressor miRNAs and inhibition of onco-miRs are considered as potentially important future therapeutic strategies [18].

As new miRNAs are still being discovered, many potential miRNAs are still understudied [2]. miRNA 3174 is one such miRNA. In the case of miR-3174, only six cancer-related studies have been published so far. MiR-3174 has been reported as a tumor suppressor in endometrial cancer [19], gastric cancer [20], bladder cancer [21] and breast cancer [22]. However, two studies suggested its role as an onco-miR in rectal cancer [23] and in hepato-cellular carcinoma [24]. To the best of our knowledge no data are available regarding the role of miR-3174 in the pathology of GBM, therefore, the present study is planned to investigate its role in GBM.

Bioinformatics analysis and sequence alignments performed in our laboratory proposed that miR-3174 can target many influential tumor-promoting genes involved in the pathobiology of GBM, including CD44, PLAU, MDM2, CDK6 and RHOA. We therefore investigated the expression, functions and targets of miR-3174 in GBM. We hypothesized that miR-3174 acts as a tumor suppressor in GBM and inhibits growth and invasion of GBM cells and tumors.

The present study reveals that miR-3174 is downregulated in GBM cell lines, GSCs and human GBM tissue specimens. Overexpression of miR-3174 inhibited the growth and invasion of GBM cells in vitro as well as in vivo GBM xenograft growth. Further, the exogenous expression of miR-3174 in GSCs inhibited their neuro-sphere formation capacity. We also confirmed through Western blots that miR-3174 inhibits the expression of the important oncogenic targets suggested by the bioinformatics analysis.

## 2. Results

### 2.1. miR-3174 Expression Is Downregulated in GBM Tissues, Cell Lines and GSCs

**Figure 1:**
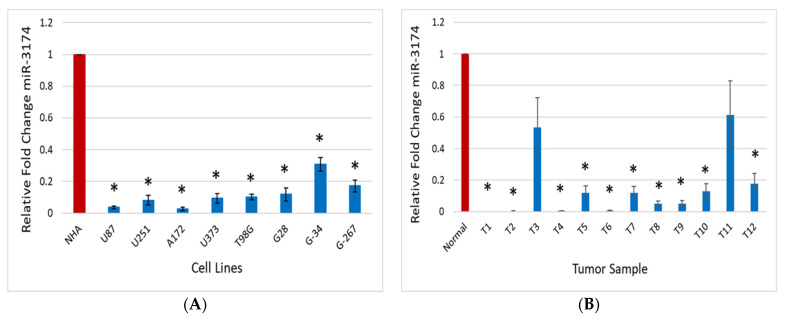
miR-3174 is downregulated in GBM cell lines, GSCs and human tumors. (**A**) miR-3174 expression was measured by qRT-PCR in a panel of GBM cell lines and GSSs and normalized to U6B small nuclear RNA gene. The results show the lower expression of miR-3174 in all GBM cell lines in comparison to NHA. (**B**) The endogenous expression of miR-3174 was evaluated using qPCR in a panel of GBM surgical tumor specimens (T) and normal brain tissue. The expression was normalized to the expression of U6B. The expression of miR-3174 was lower in GBM as compared to the normal brain tissue. The * indicates *p* ≤ 0.05.

To investigate the expression of miR-3174’s role in GBM, we analyzed the endogenous expression of miR-3174 in multiple GBM (grade 4) tumor tissues (*n* = 12), normal human astrocytes (NHA), GBM cell lines (U87, U251, A172, U373 and T98G) as well as in GSCs (G28, G34 and G267) using quantitative PCR. The experiment was carried out thrice in triplicate. The results revealed the significantly (*p* < 0.05) lower expression of miR-3174 in all the tumor tissues as compared to the normal brain tissue (Figure 1A) and in all GBM cell lines and GSCs as compared to NHA (Figure 1B). The downregulated expression of miR-3174 in all GBM cell lines, tissues and GSCs highlights that miR-3174 likely acts as a tumor suppressor in GBM. 

### 2.2. miR-3174 Inhibits Proliferation and Invasion of GBM Cells

**Figure 2:**
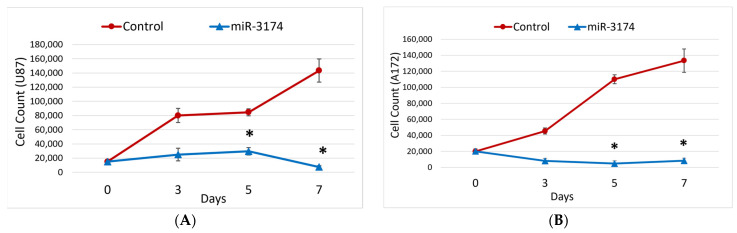

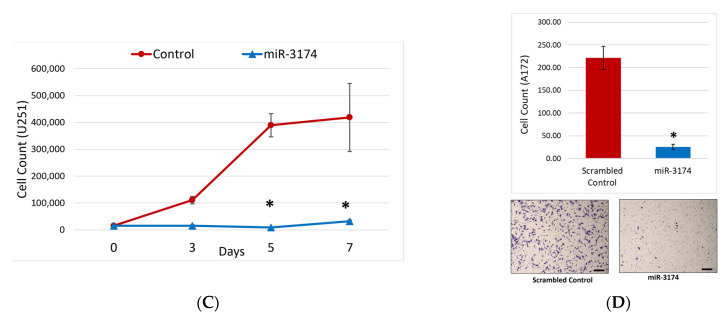
miR-3174 inhibits GBM cells proliferation and invasion. GBM cell lines were transfected with pre-miR-3174 or pre-miR-scrambled control and subsequently evaluated for cell proliferation by cell counting on the 3rd, 5th and 7th day post transfection. miR-3174 inhibited the proliferation of all the transfected GBM cell lines including (**A**) U87 (**B**) A172 and (**C**) U251 in comparison to miRNA scrambled control-transfected cells. (**D**) The effect of miR-3174 on invasion was evaluated in A172 GBM cells post transfection using transwell cell invasion assay. miRNA-3174 inhibited the invasion of GBM cells as compared to the miRNA scrambled control-transfected cell. All scale bars represent 100 µm. The * indicates *p* ≤ 0.05 in Student’s *t*-test.

To confirm the likely tumor-suppressive role of miR-3174 in GBM as indicated by its endogenous downregulation, we overexpressed miR-3174 or miR-scrambled control in GBM cell lines and assessed its effect on proliferation and invasion of GBM cells. For proliferation or growth assay, pre-miR-3174 or pre-miR-scrambled control (non-targeting stem-loop sequence used as negative control) were transfected into U251, U87 and A172 cells and cells were counted on the 3rd, 5th and 7th day post transfection. Exogenous expression of pre-miR-3174 significantly (*p* < 0.05) reduced the growth of all GBM cell lines in comparison to the cells transfected with pre-miR-scrambled control (Figure 2A–C). Further, the effect of miR-3174 on the invasion of GBM cells was also evaluated. A172 cells were transfected with either pre-miR-3174 or pre-miR-scrambled control and invasion assay was performed after 48 hrs. Experiments were carried out thrice in triplicate. Results revealed that miR-3174 significantly (*p* < 0.05) inhibited the invasion of GBM cells (Figure 2D). Altogether these data suggest that miR-3174 is a strong inhibitor of GBM cell growth and invasion.

### 2.3. miR-3174 Impedes the Sphere Formation Ability of GSCs

**Figure 3:**
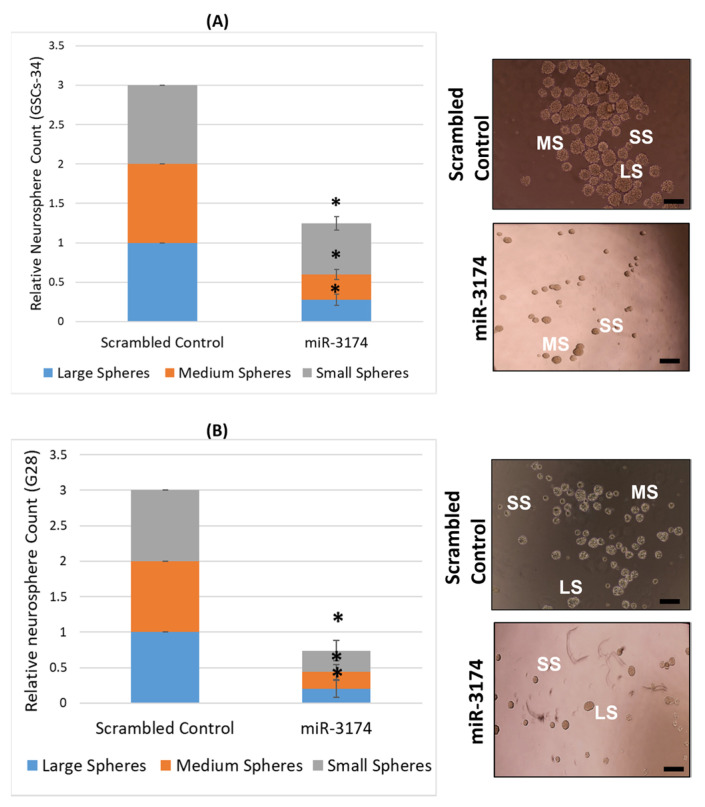
miR-3174 inhibits sphere formation or self-renewal capability of GSCs. GSC82 and GSC34 cells were transfected with either pre-miR-3174 or pre-miR-scrambled control followed by the sphere formation assay. Spheres (large, medium and small) were counted on 7th day post transfection followed by statistical analysis. miR3174 inhibited the sphere formation ability of both (**A**) GSC28 and (**B**) GSC34 in comparison to miRNA scrambled control as also visible in representative images. All scale bars represent 100 µm. The * indicates *p* ≤ 0.05.

To investigate the effect of miR-3174 on self-renewal of GSCs, the neuro-sphere formation assay was performed using two patient-derived GSCs cell lines (GSC28 and GSC34). GSCs were transfected with either pre-miR-3174 or pre-miR-scrambled and spheres were counted on 7th day post transfection. Spheres were divided into three categories (large, medium and small) as per their observed size under the microscope at 10X. The results revealed that spheres were significantly (*p* < 0.05) less in number in wells having GSCs transfected with miR-3174 in comparison to pre-miR-scrambled control transfected GSCs. Similar results were obtained for both the GSCs cell lines (Figure 3A,B). These data provide evidence that miR-3174 inhibits the self-renewal ability of GSCs. 

### 2.4. miR-3174 Negatively Regulates the Expression of Multiple Tumor-Promoting Genes

**Figure 4:**
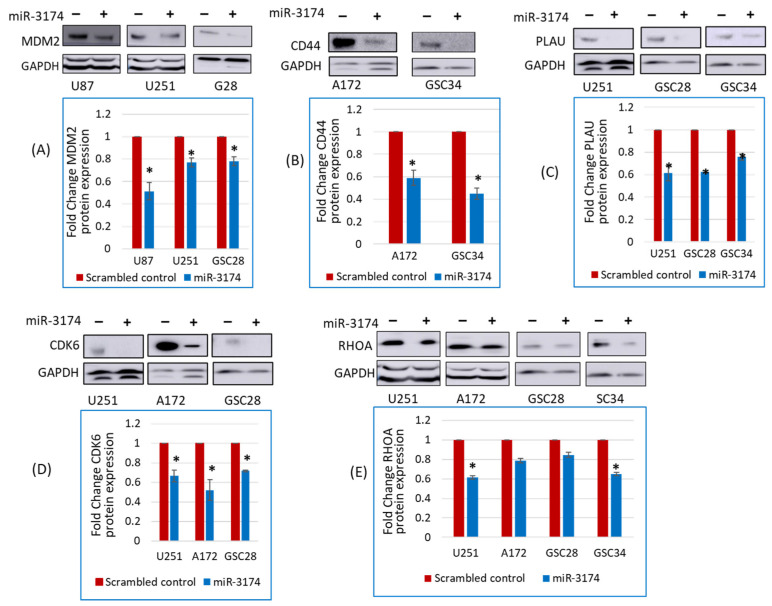
miR-3174 inhibits the expression of multiple tumor-promoting genes. GBM cell lines and GSCs were transfected with either pre-miR-3174 or pre-miR-scrambled control followed by total protein lysate collection and immunoblotting. Band intensities from immunoblot replicates were quantified, averaged and plotted as graphs, where (−) indicates cells transfected with pre-miR-scrambled control group and (+) indicates pre-miRNA-3174 transfected cells. (**A**) Representative immunoblots of CD44 and their respective GAPDH along with the bar graph. (**B**) Representative immunoblots of MDM2 and their respective GAPDH along with the bar graph. (**C**) Representative immunoblots of CDK6 and their respective GAPDH along with the bar graph. (**D**) Representative immunoblots of RHOA and their respective GAPDH along with the bar graph. (**E**) Representative immunoblots of PLAU and their respective GAPDH along with the bar graph. * indicates *p* < 0.05.

Having confirmed the tumor-suppressive effects of miR-3174 using different functional assays, we searched for target genes of miR-3174 using bioinformatics and sequence alignment tools. The tools predicted several tumor-promoting genes as a potential target of miR-3174 including CD44, CDK6, MDM2, RHOA and PLAU (Figure S1). To confirm the effect of miR-3174 on the predicted tumor-promoting genes, we investigated the effect of exogenous expression of miR-3174 on the protein expression of the mentioned tumor-promoting genes using immunoblotting. Briefly, cells were transfected with pre-miR-3174 or pre-miR-scrambled control, and total protein lysates were collected and quantified followed by immunoblotting for the predicted tumor-promoting genes. Immunoblotting results revealed that overexpression of miR-3174 significantly downregulated the protein expression of several tumor-promoting genes in different cell lines and GSCs (Figure 4 and Figure S2) including MDM2 (U87 = 0.51 fold, U251 = 0.76 fold and GSC28 = 0.78 fold; Figure 4A), CD44 (A172 = 0.58 fold and GSC34 = 0.44 fold; Figure 4B), PLAU (U251 = 0.61 fold, GSC28 = 0.62 fold and GSC34 = 0.78 fold; Figure 4C), CDK6 (U251 = 0.66 fold, A172 = 0.52 fold and GSC28 = 0.71 fold; Figure 4D) and RHOA (U251 = 0.61 fold, A172 = 0.78 fold, GSC28 = 0.84 fold and GSC34 = 0.65 fold; Figure 4E) in GBM cell lines as well as in GSCs in comparison to miRNA scrambled control. Results were normalized to untreated controls (set to 1). These data suggest that miR-3174 inhibits the expression of several tumor-promoting genes in GBM.

### 2.5. miR-3174 Suppresses GBM Growth in a Mouse Xenograft Model In Vivo

**Figure 5:**
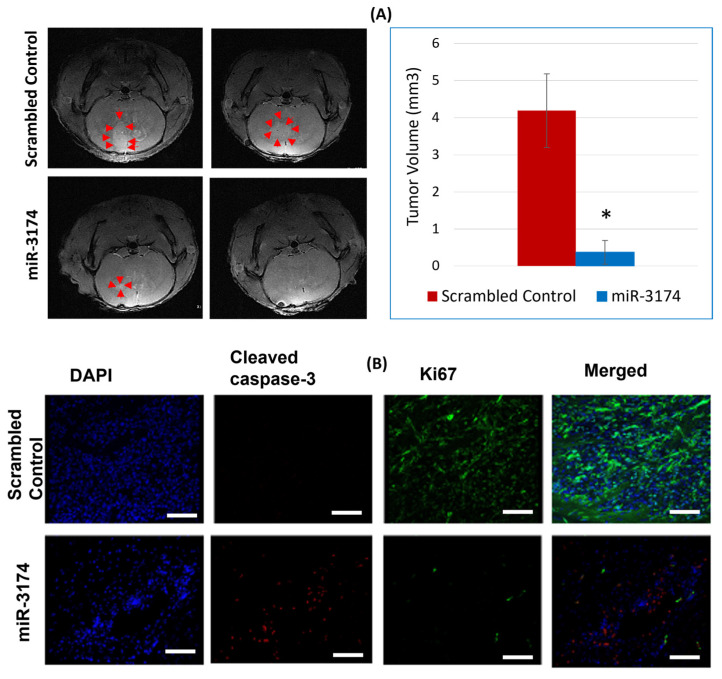
miRNA inhibits in vivo GBM xenograft growth. Pre-miR-3174 and pre-miR-scrambled control transfected U87 cells were stereotacticallty implanted into a cohort of immunocompromised nude mice. MRI was carried out on the 17th day post transfection. Tumor volumes were computed, averaged and graphed. (**A**) Representative MRI images from pre-miR-3174 and miRNA scrambled control group along with the bar graph, both showing tumor growth inhibition in miRNA-3174 treated group. The red arrow heads around the tumor demonstrating the tumor size. (**B**) Representative photomicrographs of immune-histochemical analysis of tumor tissues indicating upregulation of cleaved caspase 3 and downregulation of Ki67 in pre-miR-3174 treated group in comparison to pre-miR-scrambled control. All scale bars represent 100 µm. * indicates *p* ≤ 0.05.

We used immunocompromised nude mice to evaluate the tumor inhibitory ability of miR-3174 in an in vivo xenograft model. U87 cells were transfected with pre-miR-3174 or miRNA scrambled control and stereotactically implanted into nude mice brains (*n* = 6 for miRNA scrambled controls group and *n* = 5 for miR-3174 group). On the 17th day after cell transplantation, Magnetic Resonance Imaging (MRI) was conducted, and the tumor volumes were quantified and averaged. miR-3174 significantly (*p* < 0.05) reduced the tumor volume of U87 xenografts in nude mice as compared to the miRNA scrambled control. (Figure 5A). These experiments support the tumor-suppressive abilities of miR-3174 in an in vivo setting.

#### miR-3174 Promotes Cell Apoptosis and Inhibits Proliferation of Tumors In Vivo

To evaluate whether the tumor-suppressive role of miR-3174 in GBM in vivo is mainly induced by enhanced apoptosis or inhibition of proliferation. Immunohistochemical analysis was performed using both apoptotic (cleaved caspase 3) and proliferation marker (Ki67). The results revealed that miR-3174 exerts its tumor-suppressive effect by inhibiting proliferation and enhancing apoptosis, as evident from the downregulation of Ki67 and upregulation of cleaved caspase in the miR-3174 treated group as compared to the scrambled control group (Figure 5B). Taken together these results suggest that miR-3174 is a strong inducer of apoptosis and inhibitor of proliferation in vivo.

## 3. Discussion

In the present study we uncovered the role of miR-3174 in GBM for the first time. We demonstrated that miR-3174 acts as a tumor suppressor in GBM and has strong potential to inhibit tumor growth and invasion. miR-3174 was downregulated in several GBM tissues, cells lines and GSCs. Further we also revealed that miR-3174 functions by targeting multiple tumor-promoting genes. 

A tumor-suppressive role of miR-3174 has been reported in ovarian cancer, hepatocellular carcinoma and serous ovarian carcinoma and a few studies have reported its role as an onco-miR [19,20,21,22,23,24]. However, the role of miR-3174 in GBM has not been reported before.

The downregulation of miR-3174 in various cell lines and in tumors as compared to their normal counterparts highlighted the potential tumor-suppressive role of miR-3174 in GBM and prompted us to conduct a more in-depth study. All the functional assays and the molecular and in vivo study confirmed this notion. Further, we also demonstrated that miR-3174 downregulates multiple tumor-promoting genes (CD44, MDM2, CDK6, RHOA and PLAU) and all of these have been reported to have an important role in the development of various malignancies, including GBM [25,26,27,28,29,30]. We have not only studied miR-3174 for the first time in GBM but have also confirmed several oncogenic targets which were not identified or confirmed before in relation to miR-3174. 

The cluster of differentiation (CD44) is a complex transmembrane adhesion glycoprotein and considered an important constituent of extracellular matrix. CD44 expresses on different types of cells including cancer cells. It is encoded by 19 exons in humans. The ten constant exons encode CD44 in its standard form (CD44s). The CD44 variant isoforms (CD44v1-CD44v10) are produced by alternative splicing and have any combination of the remaining nine variant exons and the ten constant exons [31,32]. Eibl et al. analyzed the expression of CD44 and its splice-variants in primary human brain tumors and in GBM derived cell lines. All brain tumors showed the expression of the most ubiquitous isoform CD44s. Moreover, all glioblastomas expressed CD44 variants (CD44v3–CD44v10) whereas expression in astrocytomas WHO grade I, II and III could only be detected in only 50 % of the tumor samples. This indicates the role of variant isoforms of CD44 in tumor progression [33]. It is also an established surface biomarker for cancer stem cells. It plays an important role in epithelial to mesenchymal transition (EMT), tumor initiation and development. Further, it also has a significant role in invasion and metastasis of several tumors [34]. Dysregulation of CD44 is associated with multiple cancers including lung cancer [35], breast cancer [36], osteosarcoma [37] colorectal cancer [38] and glioma [39] etc. Moreover, a recent report has shown that depletion of CD44 in GBM cells induces senescence and inhibits growth and stemness [40].

Murine double minute 2 (MDM2), another target of miR-3174, is a tumor-promoting gene. Overexpression/amplification of MDM2 has been observed in many cancers including GBM [41]. MDM2 negatively regulates the important tumor suppressor P53. Hence overexpression of MDM2 inhibits cell cycle arrest and apoptotic activity of P53. Targeting MDM2 is also considered as an important strategy to revitalize p53 function and improve treatment outcomes [42]. Moreover, inhibition of MDM2 has also been shown to radiosensitize cancer cells [43].

Cyclin dependent kinase 6 (CDK6) is a frequently overexpressed gene in multiple cancers. A study has also shown upregulation of CDK6 in 44% of GBM as compared to the matched normal tissue [27]. It facilitates G1 to S cell cycle transition and certain cancer cells require CDK6 for proliferation [44]. Inhibition of CDK6 has also been shown to enhance glioma sensitivity to chemotherapy [44]. A study published in the recent past also demonstrated that inhibition of CDK6 palbociclib (specific CDK4/CDK6 inhibitor) inhibited proliferation and epithelial to mesenchymal transition of proneural GSCs [45]. The treatment also improved the survival of established intracranial xenografts of a proneural GSC line [46]. Consequently, CDK6 represents a promising target for anti-cancer therapy.

RHOA, also referred to as Ras homolog family member A, is a member of the Rho family of small GTPases and a key regulator of the invasion and migration of glioblastoma cells. Like other targets of miR-3174, RHOA is also an important controller of cellular proliferation and metastasis [30]. Chau et al. has reported that RHOA/RHO kinases regulate GBM cells proliferation and migration via interaction with the TGF- and ERK-signaling pathways [47]. It has also been suggested that inhibitors of RHO GTPase signaling may improve the survival for GBM patients if given in combination with standard therapy [30]. 

Plasminogen activator urokinase (PLAU) is a serine protease that converts plasminogen to plasmin. Besides being involved in a number of physiological processes, PLAU is also involved in angiogenesis and metastasis of tumors, making them more aggressive and therapy resistant [29]. A recent study evaluated the prognostic significance of PLAU expression in glioma using online databases. The study reported that PLAU is associated with poor prognosis and survival in glioma patients. Moreover, the co-expression network analysis revealed that PLAU is involved in signaling pathways related to inflammation and tumorigenesis [48]. Some previous studies have also shown that up-expression of PLAU was associated with glioma growth, invasion, and angiogenesis [49,50]. 

Keeping in view the diverse role of the miR-3174 targeted tumor-promoting genes in the pathology of cancers and specifically in GBM, it can be inferred that miR-3174 has a potential to target multiple signaling pathway in GBM and can be used as tumor suppressor therapeutic agent in GBM. Altogether, our data show that miR-3174 is a new potent tumor suppressor miRNA that acts by targeting several tumor-promoting genes in GBM.

## 4. Materials and Methods

### 4.1. Tumor Specimens and Cell Lines

Glioblastoma patient tissue samples were obtained from the University of Virginia Bio-repository and Tissue Specimen Facility as per reviewed and approved procedures of the Institutional Review Board. Glioblastoma cell lines U87, U251, A172, U373 and T98G were procured from ATCC (Manassas, VA, USA). GBM stem cells GSC28 and GSC34 were a generous gift from Drs. Erik P. Sulman and Krishna Bhat (MD Anderson Cancer Center) and Dr. Jakub Godlewski, Harvard Medical School respectively. The GSCs were isolated from patient surgical specimens and characterized for stem-cell markers, pluripotency, in vivo tumorigenesis, self-renewal, and neurosphere formation. U87 cells were grown in MEM supplemented with 1 mM sodium pyruvate, 0.15% (*w*/*v*) sodium bicarbonate, 1% non-essential amino acids and 10% FBS; U251 cells were grown in RPMI with 5% FBS; A172, cells were grown in DMEM with 4.5 g/L glucose supplemented with 10% FBS; U373 cells were also grown in DMEM with 4.5 g/L glucose supplemented with HEPES (1M) and 10% FBS. T98G cells were grown in MEM supplemented with 10% FBS. While GSCs were grown in Neurobasal Media supplemented with L-glutamine (0.5 mM), N2 and B27 supplements (0.5×), human recombinant bFGF and EGF (50 ng/mL) (R&D Systems, Minneapolis, MN, USA). All the other reagents and media were from Thermo Fisher Scientifics (Waltham, MA, USA). All cells were cultured in cells specific media and maintained at 37 °C in an incubator with 5% CO_2_ and 20% O_2_. 

### 4.2. Reagents

Transfection reagent Lipofectamine RNAiMAX, Opti-MEM I Reduced Serum Media, Pre-miR-3174 and pre-miR-scrambled control all were purchased from Thermo Fisher Scientific (Waltham, MA, USA).

### 4.3. Quantitative RT-PCR

All collected tissue samples were homogenized and then lysed using TRIzol reagent. Total miRNA was then extracted from each sample including cell lines using the miRNeasy kit (Qiagen, Chatsworth, CA, USA, Cat. No. 217084). Subsequently, cDNA was synthesized using the miRCURY LNA RT Kit (Qiagen, Cat. No. 339340). Quantitative PCR was performed using company designed primer assays for miR-3174 and U6B (housekeeping gene) with the miRCURY LNA SYBR Green PCR Kit (Qiagen, Cat. No. 339346). 30 ng of cDNA was used for each PCR reaction. The conditions for the qPCR were the same as described in the kit protocol. Expression analysis was conducted as explained previously [51]. 

### 4.4. Cell Growth Assay

All GBM cell lines were plated in 6-well plates at a density of 15,000–20,000 cells/well. After 24 h of plating, the cells were transfected with either pre-miR-3174 (30 nM) or pre-miR-scrambled control (30 nM) using Lipofectamine RNAiMAX transfection reagent. Before the transfection, complete growth media in all the wells was replaced with Opti-MEM I Reduced Serum Media (ThermoFisher Scientific). After four hours of transfection, all the transfection reagents in the wells were replaced with complete growth media. Then cells were trypsinized and counted using hemocytometer on day 3 (72 h), 5 and 7 post transfection as previously described [3].

### 4.5. Invasion Assay

GBM A172 cells were plated at a density of 150,000 cells per well in a 6-well plate and transfected 24 h later with either pre-miR-miR-3174 or pre-miR-scrambled control. After 72 h, the cells were re-suspended in media containing 0.1% FBS and seeded in (100,000 cells/500 µL) chambers pre coated with collagen IV and Invasion assay was performed as previously described [3]. The images were analyzed using ImageJ Software version 1.53t (National Institutes of Health, Bethesda, MD, USA).

### 4.6. Glioma Stem Cell Sphere Formation Assay

GSCs (GSC28 and GSC34) were grown in Neurobasal media containing FGF and EGF and treated and transfected with either pre-miR-3174 or pre-miR-scrambled control and incubated for 72 h. After 72 h the cells were dissociated into single cell suspension using accutase dissociation buffer (Sigma, Rockville, MD, USA) and 1000 single cells were transferred to 24-well plates in triplicates and incubated at 37 °C for 7 days. Secondary neurospheres containing more than 30 cells were quantified into three categories, i.e., large, medium and small spheres as per their visible size.

### 4.7. Immunoblot Analysis

To confirm the effect of miR-3174 on its predicted targets immunoblot analysis was performed. Briefly, GBM and GSC cell lines were transfected with either pre-miR-3174 or pre-miR-scrambled control. After 48 h of transfection total cell lysate was extracted and protein concentration of each sample was determined using Bradford protein assay as per the established protocol. Immunoblotting was performed according to standard protocols. The following antibodies were used for the detection of protein targets: MDM2 (OP115; Millipore Sigma, Rockville, MD, USA), CD44 (3570S, Cell Signaling Technology, Danvers, MA, USA), HMGA2 (8179S; Cell Signaling Technology), RHOA (67B9; Cell Signaling Technology, Danvers, MA, USA) CDK6 (D4S8S; Cell Signaling Technology, Danvers, MA, USA), PLAU (17968-1-AP; proteintech) and GAPDH (sc-5284, Santa Cruz Biotechnology, Dallas, TX, USA). Each immunoblot was also immune stained with GAPDH (Santa Cruz Biotechnology, Dallas, TX, USA) as a loading control. All antibodies were used at a dilution of 1:250 except CD44 and GAPDH which were used in 1:1000 dilutions. Band intensities were quantified using image J. Double bands in case those of GAPDH were too close. For perfect normalization and quantification, it was made sure during quantification that the selection of area around the band remained close and just around the specific band of interest.

### 4.8. In Vivo Experiments

The effect of miR-3174 on in vivo tumor growth and survival was tested in an intracranial xenograft model. Briefly, GBM (U87) cells were transfected with pre-miR-3174 or pre-miR-scrambled control. After 24 h, the cells were collected, and 3 × 10^5^ cells were stereotactically implanted in the striata of immunodeficient mice (*n* = 6 for miR-scrambled control group and *n* = 5 for pre-miR-3174). Brains were scanned with magnetic resonance imaging on a 7 Tesla Bruker/Siemens ClinScan small animal MRI on the 17th day after cell transplantation. Tumor volumes were quantified using OsiriX Lite software (Version 12.5.2, Pixmeo Sarl, Bernex, Switzerland). 

### 4.9. Immuno-Histochemical Analysis

Immunohistochemistry was conducted to analyze the protein expression of apoptotic marker active caspase 3 and proliferation marker Ki67 in GBM brain tumor xenografts. Briefly, the brains were removed, fixed in PFA, submerged in 30% sucrose solution, frozen in OCT media, cryo-sectioned (10 µm) and then subjected to immuno-staining using following primary and secondary antibodies. Rabbit cleaved caspase 3 (9661S; Cell Signaling Technology, Danvers, MA, USA), Ki67 (9449S; Cell Signaling Technology, Danvers, MA, USA), Alexa flour 555 donkey anti rabbit (A31572; Invitrogen, Waltham, MA, USA) and Alexa flour 488 donkey anti mouse (A21202; Invitrogen) were used. Images were taken on Zeiss LSM 880 confocal microscope at 20× magnification.

### 4.10. Statistical Analysis

All experiments were conducted in at least three replicates. Where applicable, two group comparisons were analyzed using a Student’s *t*-test, and the *p*-values were computed.

## Data Availability

Not applicable.

## Supplementary Materials

The supporting information can be downloaded at: https://www.mdpi.com/article/10.3390/ijms24119326/s1.
