# Supplementary material for: miR-3174 Is a New Tumor Suppressor MicroRNA That Inhibits Several Tumor-Promoting Genes in Glioblastoma
Source: Int J Mol Sci. 2023 May 26;24(11):9326. doi: 10.3390/ijms24119326 (PMC10253284; doi:10.3390/ijms24119326)
Supplement: Supplementary file 1 [file ijms-24-09326-s001.zip › ijms-2408172-supplementary.pdf]

Supplementary Material

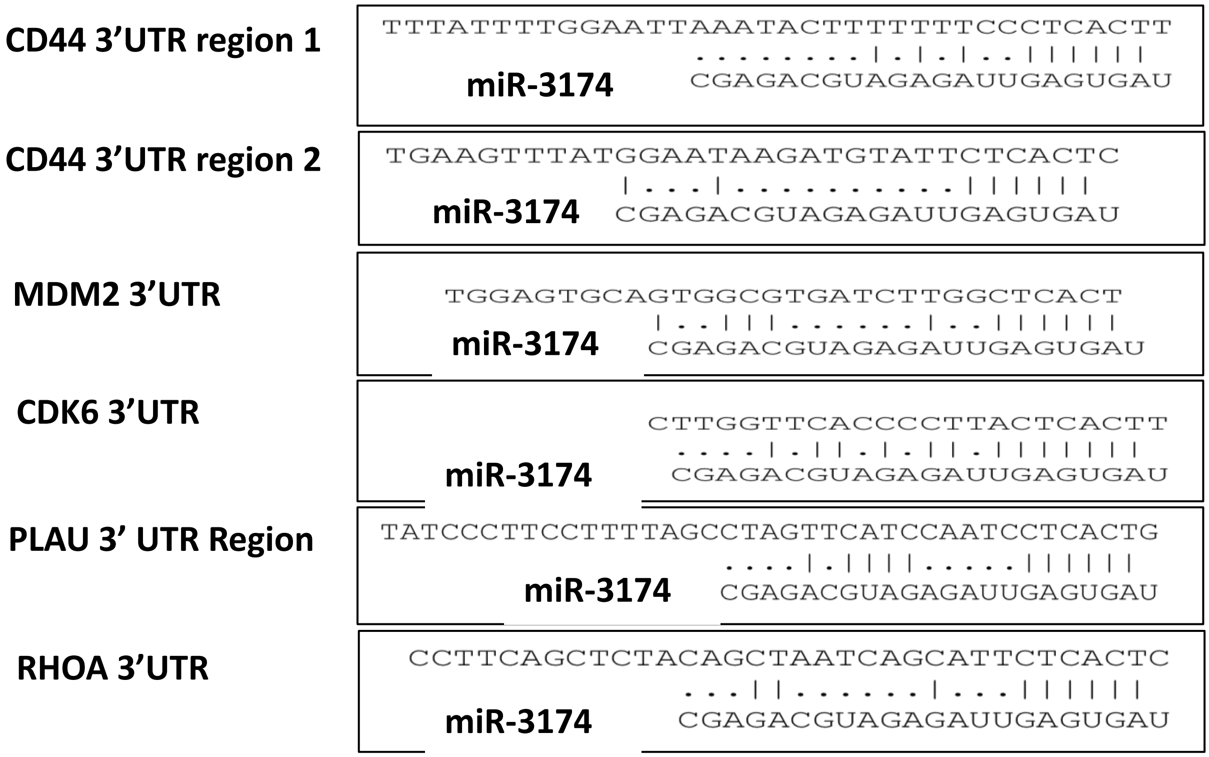

Figure S1. The miR-3174 alignment to each target gene. The alignment is based on Target Scan (online) and our PAR-CLIP analysis (unpublished)

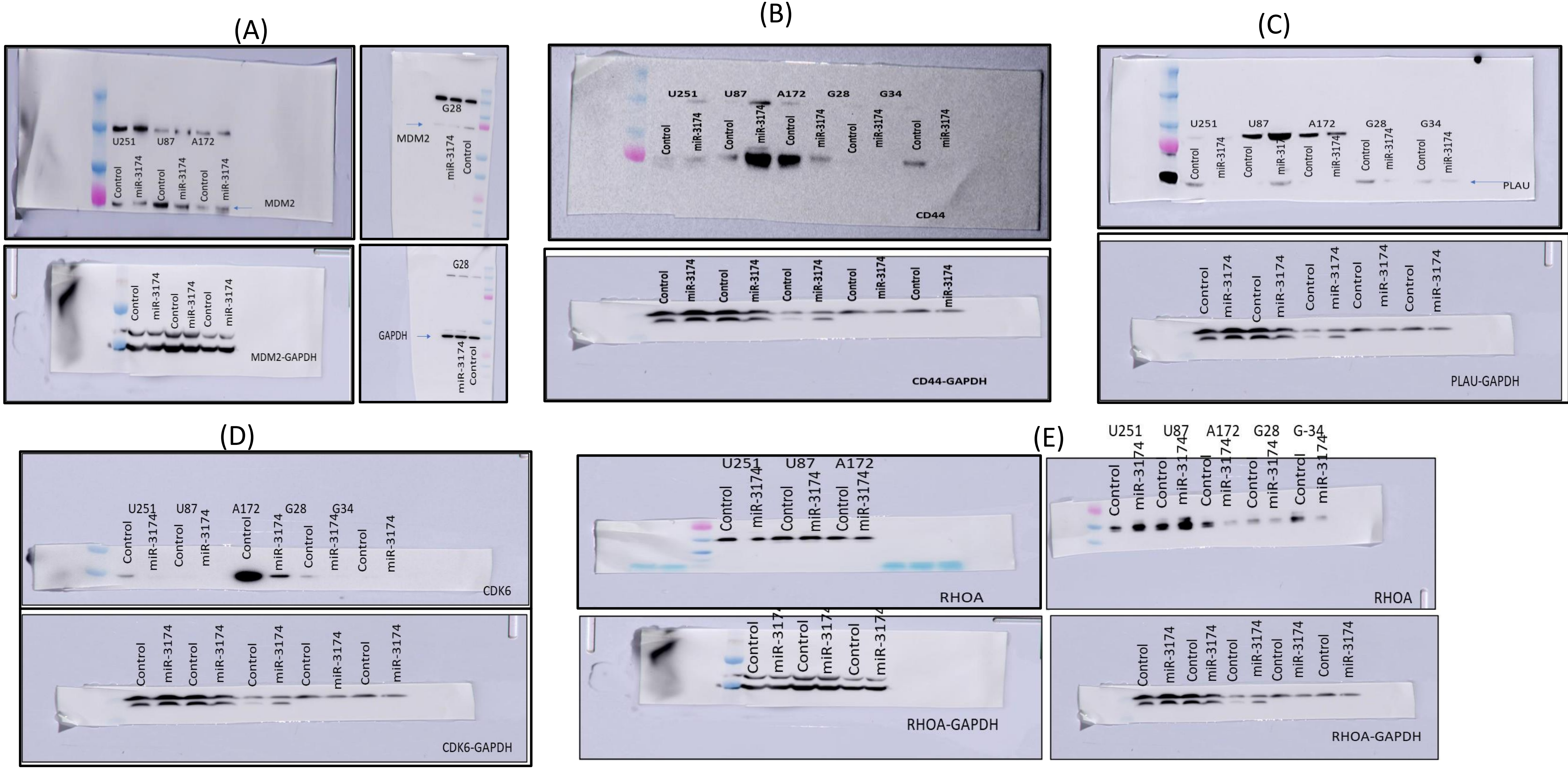

Figure S2. Representative raw immunoblots. (a) Immunoblot of MDM2 and associated GAPDH. (b) Immunoblot of CD44 and associated GAPDH. (C) Immunoblot of PLAU and associated GAPDH. (D) Immunoblot of CDK6 and associated GAPDH.(E) Immunoblot of RHOA and associated GAPDH. Cell lysates were collected from GBM cell lines and GSCs that were transiently transfected with either microRNA scrambled control (Control) or miR-3174.
